# Disseminated cutaneous *Actinomyces bovis* infection in an immunocompromised host: case report and review of the literature

**DOI:** 10.1186/s12879-022-07282-w

**Published:** 2022-03-29

**Authors:** Flávia Cunha, David Lopes Sousa, Luís Trindade, Vítor Duque

**Affiliations:** 1 Infectious Diseases Department, Centro Hospitalar e Universitário de Coimbra, Coimbra, Portugal; 2 Internal Medicine Department, Centro Hospitalar e Universitário de Coimbra, Coimbra, Portugal; 3 Faculty of Medicine, University of Coimbra, Coimbra, Portugal

**Keywords:** Actinomycosis, *Actinomyces bovis*, Case report, Immunosuppression, Ulcerous colitis

## Abstract

**Background:**

Actinomycosis is an uncommon endogenous bacterial infection caused by *Actinomyces* species, characterized by the development of abscesses, tissue fibrosis, and fistulisation. It remains a diagnostic challenge, due to its similarities with diverse aetiologies’ presentation, such as neoplasms, tuberculosis, or fungal infections. *Actinomyces bovis* is a microorganism rarely reported as a cause of human disease. Cutaneous involvement is sporadic. In this case, *Actinomyces bovis* was responsible for disseminated cutaneous disease in an immunosuppressed patient.

**Case presentation:**

We report the case of a 69-year-old female with multiple skin masses, under immunosuppressive therapy due to ulcerative colitis. Imaging exams were compatible with multiple cutaneous abscesses in the cervicofacial region and limbs. *Actinomyces bovis* was isolated in culture after abscess drainage. Antimicrobial therapy with parenteral penicillin G and oral amoxicillin was administered for 6 months, with complete resolution of cutaneous lesions and no relapse of the infection.

**Conclusions:**

Considering actinomycosis as a possible diagnosis in the presence of subacute/chronic recurrent mass-like cutaneous lesions, especially in the setting of immunosuppression, may reduce the burden associated with delayed diagnosis and incorrect treatment and provide better outcomes and improvement of patient’s quality of life.

## Background

Actinomycosis is an uncommon sub-acute or chronic suppurative, granulomatous infection caused by bacteria from the genus *Actinomyces*. These strict or facultative aerobic or microaerophilic filamentous Gram-positive bacilli are commensal microorganisms of human oral, gastrointestinal and genital mucosa [1–4]. There are currently 49 species and 2 subspecies identified, with 26 species being described as a cause of disease in humans [5]*.* The most common etiologic agents are *A. israelii, A. naeslundii, A. odontolyticus, A. viscosus, A. meyeri, and A. gerencseriae* [3, 5]. Actinomycosis manifests as purulent, mass-like lesions, involved by tissue fibrosis, with occasional fistulae development and drainage. It can affect virtually all tissues and organs of the human body and is capable of progress across tissue boundaries. Cervicofacial (head and neck) infections are the most prevalent form, counting up to 40–60% of the reports [3, 5, 6]. Cutaneous/soft tissue involvement is rare and may result from trauma or spread from different organs [3, 5]. The diagnosis of actinomycosis may be difficult, as its presentation can mimic other diseases, namely solid tumours, active *Mycobacterium tuberculosis* infections, nocardiosis, fungal infections, infarctions (in the lungs), or other granulomatous diseases [3, 5].

Here, we describe a case of cervicofacial actinomycosis with disseminated cutaneous involvement caused by *Actinomyces bovis* in a patient under pharmacological immunosuppression due to ulcerative colitis.

## Case presentation

**Fig. 1:**
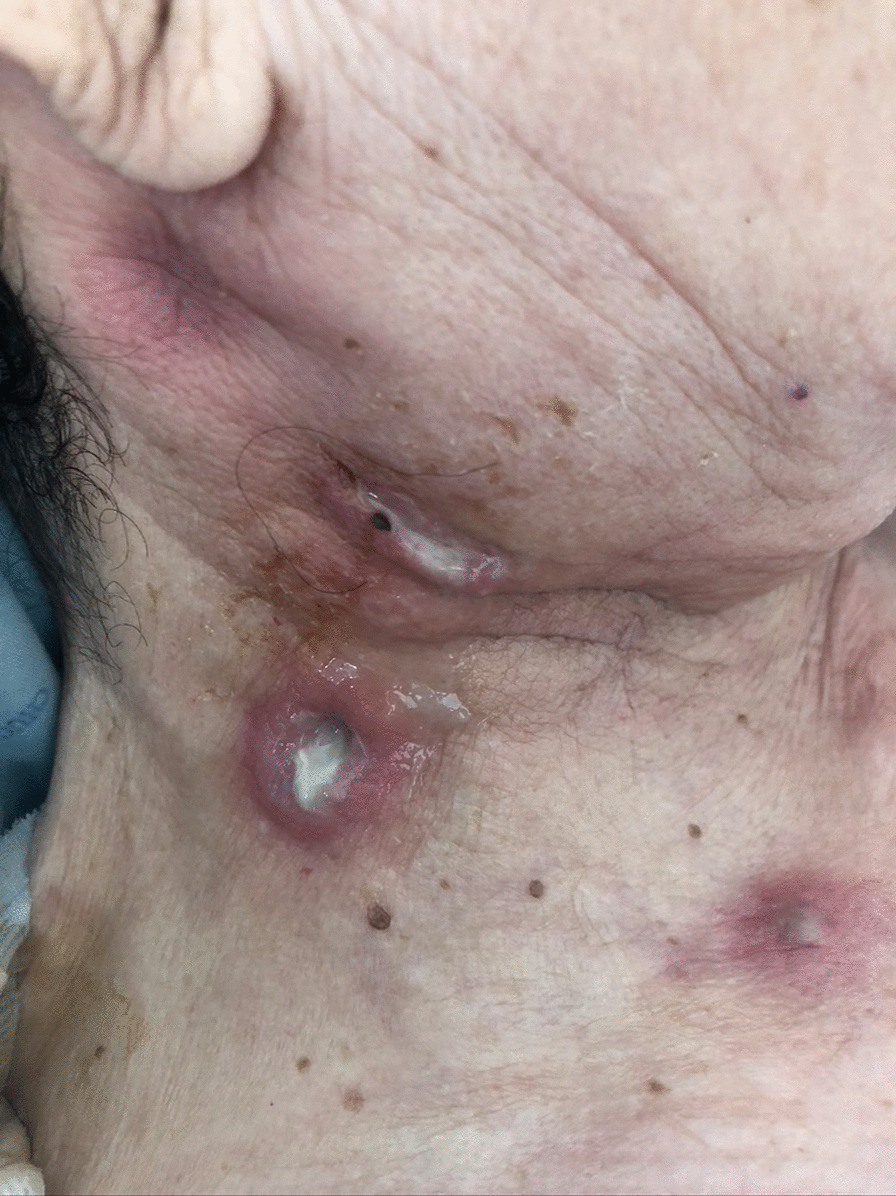
Cervical lesions with drainage of purulent material

**Fig. 2:**
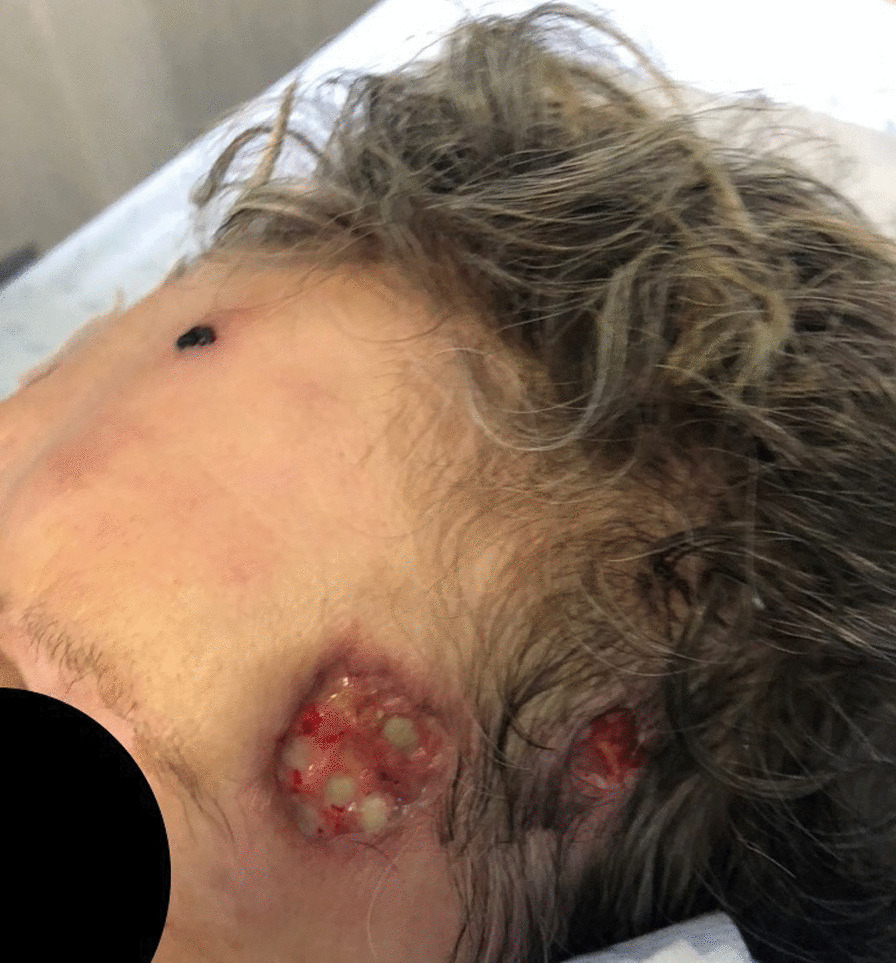
Forehead lesions with presence of yellowish grains

**Fig. 3:**
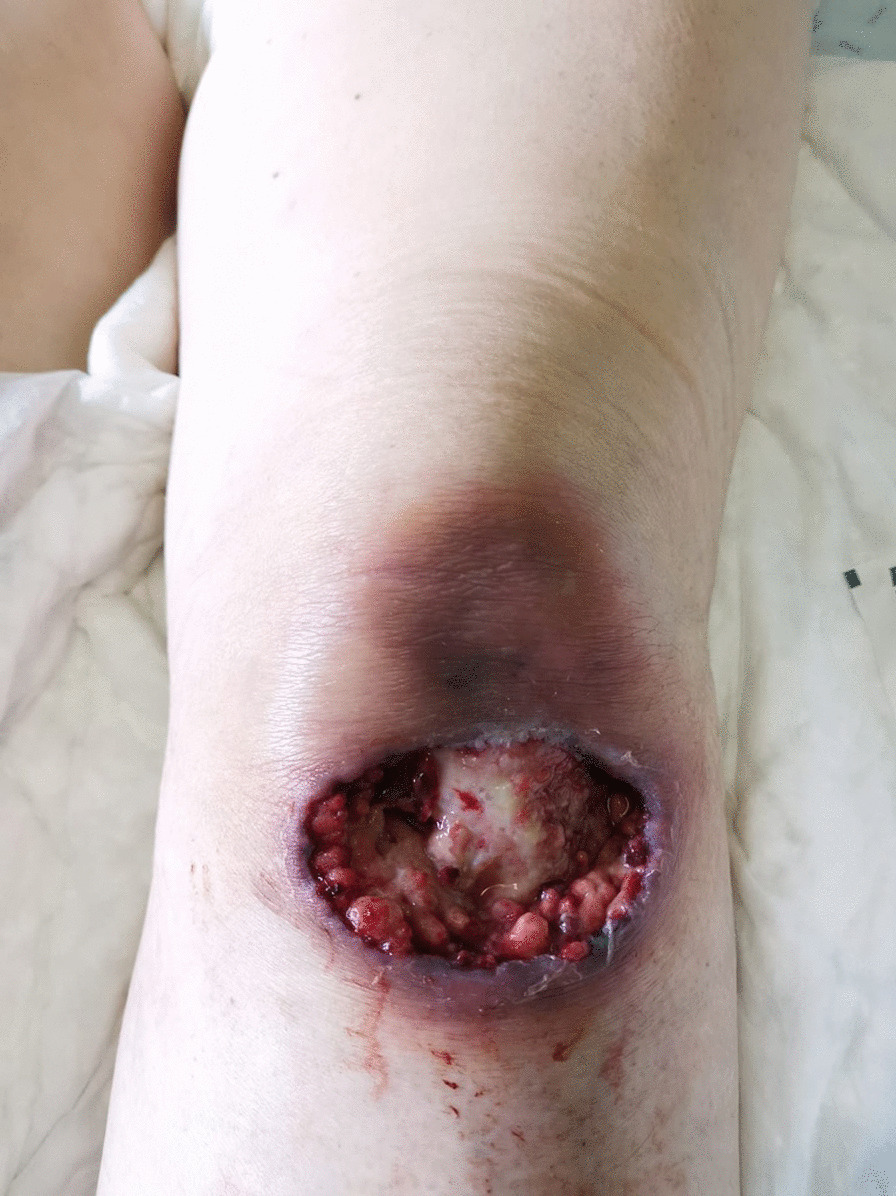
Knee abscess with loss of deep tissue integrity

We describe the case of a 69-year-old female admitted to the Infectious Diseases department with a 3-week history of multiple cutaneous masses. She denied fever, chills, headache, neurological deficits, or weight loss. She reported recurrent falls weeks earlier due to antidepressant-related dizziness periods, resulting in several skin wounds on her face and limbs. The first noticed lesion was a tender nodule in the pre-auricular area, which led her to contact her general practitioner, who prescribed doxycycline 100 mg bid for 1 week. As there was a progression of the lesions, with extension to the parotid and cervical regions, she was then medicated with levofloxacin 500 mg qd, without clinical response. She also recalled being in close contact with her pet cat (namely being licked and scratched by him) prior to the appearance of the lesions and denied contact with other animals. Her medical history was remarkable for a history of treated latent tuberculosis, persistent depressive disorder medicated with mirtazapine 30 mg qd and lorazepam 2.5 mg qd, and ulcerative colitis medicated with azathioprine 100 mg qd and mesalazine 3 mg qd. On physical examination, numerous tender cutaneous masses were visible on the right cervical region, frontal area, forearms, and knees bilaterally, some of them with a spontaneous discharge with the presence of yellow grains (Figs. 1, 2) and loss of integrity of deep skin layers (Fig. 3).

**Fig. 4:**
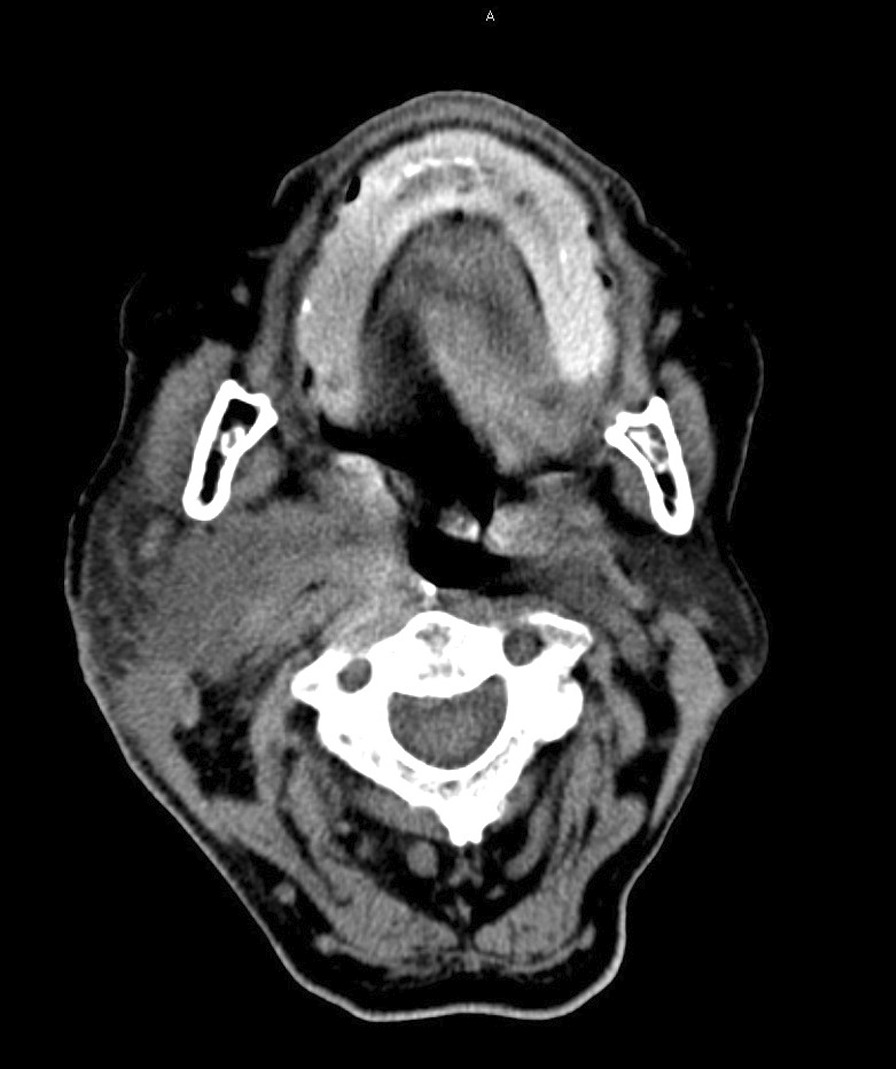
Cervical lesion observed on cervical CT scan

Laboratory results showed a normocytic anaemia (haemoglobin 8.2 g/dL, mean corpuscular volume 89.7 fL) and elevated inflammatory markers (C reactive protein 25.59 mg/dL and erythrocyte sedimentation rate 83 mm/h). Serological tests were negative for human immunodeficiency virus (HIV), hepatitis B and C, syphilis, and *Bartonella henselae.* Cervical computed tomography (CT) showed a 43 × 55 mm cystic lesion medial to the right parotid, as well as smaller abscesses in the submandibular area and posterior pharynx wall (Fig. 4). Soft tissue ultrasound confirmed the presence of abscesses in forearms and knees, with the larger one measuring 79 mm in the major axis. Turbid pus was drained percutaneously from both cervical and forearm abscesses. Polymerase chain reaction (PCR) testing for *Mycobacterium tuberculosis complex,* acid-fast staining, mycobacterial and fungi culture of this material were negative, as well as blood cultures. Gram staining revealed Gram-positive rods, surrounded by polymorphonuclear leucocytes. Bacteriological culture of the pus from the forearm in CDC Anaerobe Agar with 5% Sheep Blood, kept at 37 ºC, isolated *Actinomyces bovis*.

Histopathological examination of skin biopsy (performed 2 weeks after antibiotic treatment were started) revealed unspecific inflammatory infiltrates comprising macrophages and neutrophils, without evidence of neoplastic infiltration.

To rule out other foci of actinomycosis, head, thoracic and abdominal CTs were performed, with no further lesions detected. Positron emission tomography (PET) scan revealed generalized hypermetabolic cutaneous lesions in the forehead, submandibular area, neck, forearms, and knees bilaterally, as well as cervical and axillary adenopathies.

**Fig. 5:**
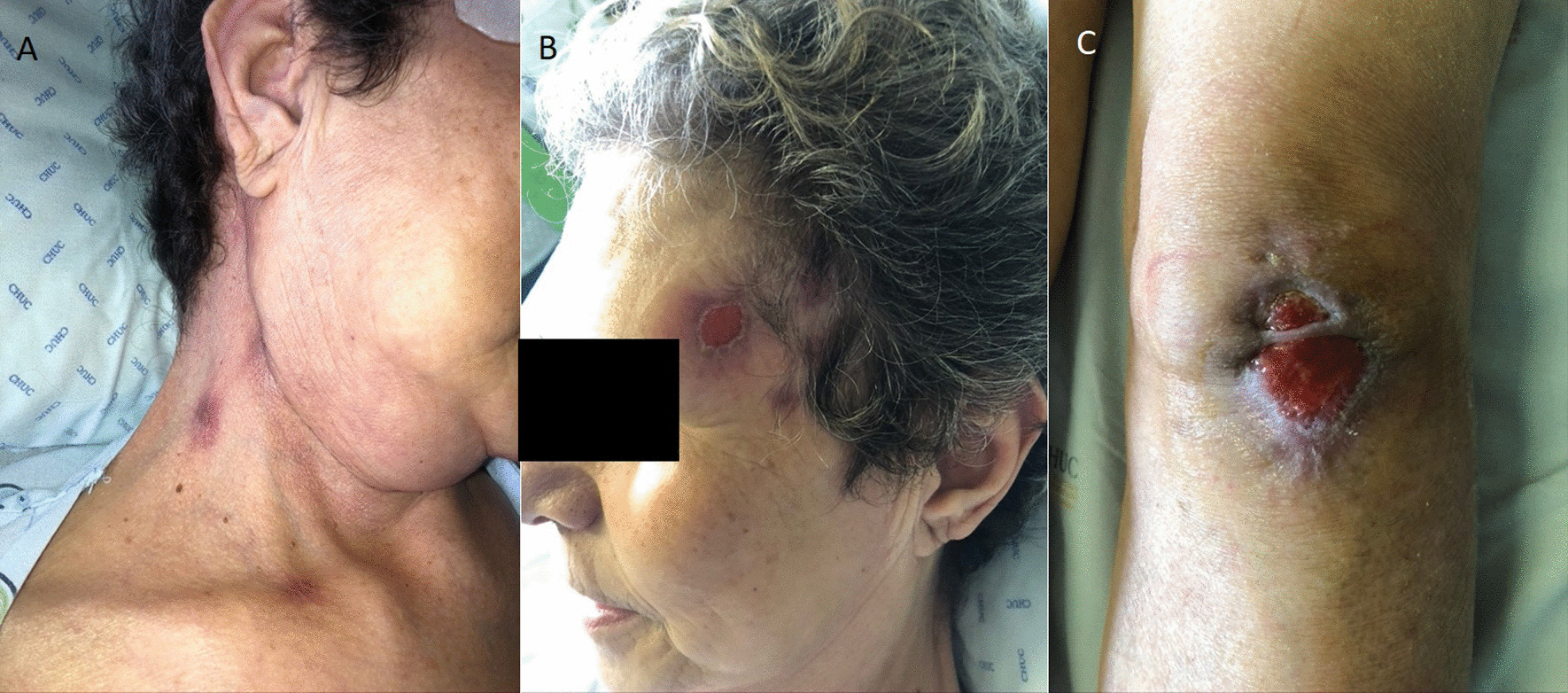
Improvement of the cervical (**a**), forehead (**b**) and knee (**c**) lesions after 6 weeks of antibiotic treatment

**Fig. 6:**
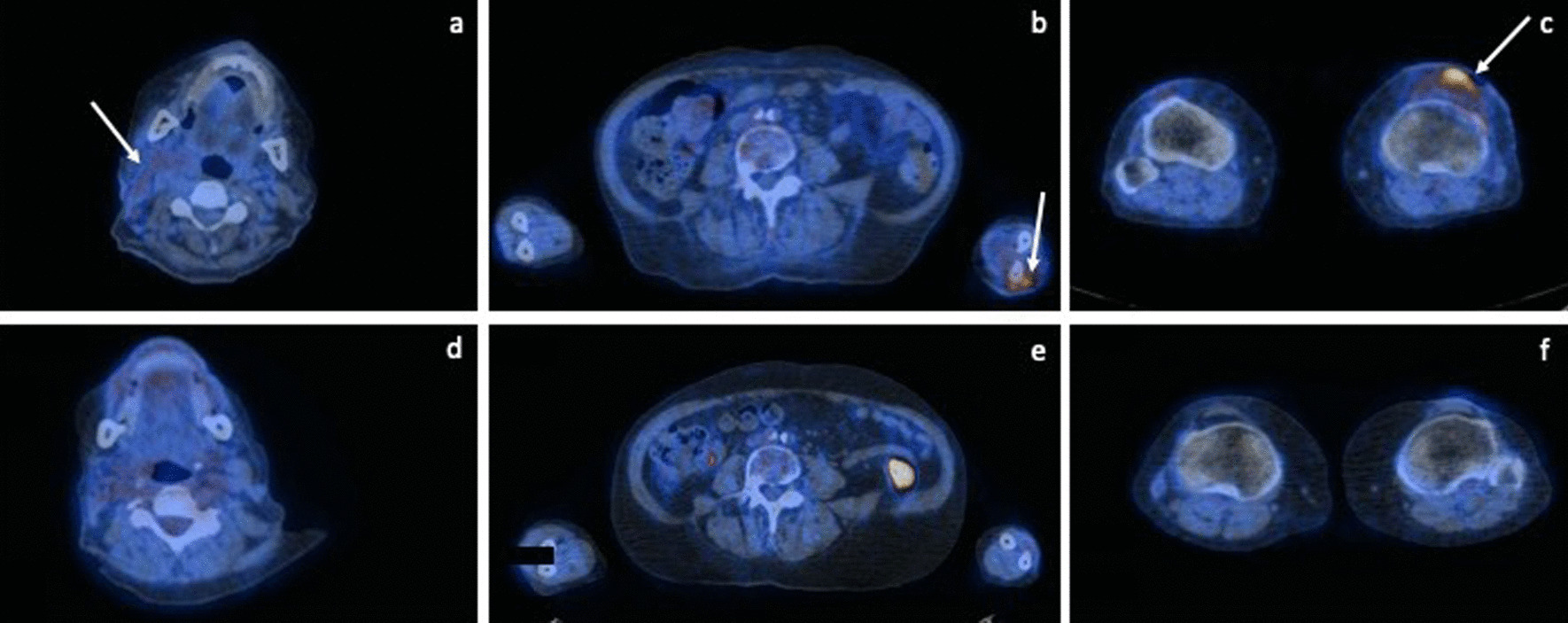
PET scan images from cervical, forearm and knee lesions at the diagnosis (**a**–**c**) and after antibiotic treatment (**d**–**f**)

Parenteral penicillin G 3 million units 4-h was given for 6 weeks, with significant reduction of the abscesses and recovery of skin integrity (Fig. 5). The patient completed 6 months of treatment with oral amoxicillin 1 g tid, with complete resolution of cutaneous lesions supported by follow-up PET scan, not requiring additional surgical correction (considering the significant deep tissue damage due to the infection at diagnosis); Fig. 6 shows the PET scan at the diagnosis and after the treatment. No recurrence of the disease was observed after 18 months of follow-up.

## Discussion and conclusions

Actinomycosis is a rare, frequently underestimated infectious disease caused by bacteria from the *Actinomyces* genus, with an estimated annual incidence of 1/300,000 persons in the 1970s in the Cleveland area [1, 3]. It has been described worldwide, but with a higher proportion of cases in developing countries, as a result of poor oral hygiene and dental care, as well as limited access to healthcare services and antibiotic treatments [3]. Misdiagnosis could be a possible explanation for the low reported incidence of actinomycosis, considering its vast differential diagnosis—solid tumours, active *Mycobacterium tuberculosis* infections, nocardiosis, fungal infections, pulmonary infarctions, or granulomatous diseases [1, 5, 6].

Actinomycosis is considered mostly an endogenous infection, given that these microorganisms are typically part of the bacterial flora of the human mucous membranes and become pathogenic if the mucosal barrier is disrupted (due to trauma, surgery, or lesion due to foreign bodies) [6]. It may occur in patients of all ages, but with lower incidence in younger and older age groups. Also, it affects more frequently men than women, with male-to-female ratios varying with age [3, 4].

These infections are described as involving the cervicofacial, thoracic, and abdominopelvic regions and the central nervous system (CNS). Cervicofacial actinomycosis usually presents as chronic, painless, or occasionally painful soft-tissue swelling of the submandibular or perimandibular region, draining sinus tracts with sulfur granules, difficulties in chewing, and chronic/relapsing course of the infection, even after several antibiotic courses [3, 5]. Possible risk factors for cervicofacial and oral actinomycosis include dental procedures, caries, gingivitis, chronic tonsillitis, periodontal disease, otitis or mastoiditis, possibly diabetes, and immunosuppression, malnutrition, and local tissue injuries by tumours, surgery, irradiation, or trauma [3]. Cutaneous actinomycosis is usually a secondary disease, resulting from either contiguous extension from a deeper source of infection or hematogenous spreading from an extracutaneous (often pulmonary) origin [7]. In our patient, considering her description, there was cervical involvement at first, with subsequent development of distant cutaneous foci; these were compatible with previous wounds sites as a result of her falls. Considering the negative blood cultures and exclusion of deeper visceral involvement by imaging studies, the diagnosis of ‘isolated cutaneous disseminated actinomycosis’ was assumed. However, it is relevant to notice that cultures (from different types of samples) are negative in about 50% of the cases [5], especially if the samples were taken after the onset of antimicrobial therapy; in spite of negative blood cultures, we cannot completely rule out the possibility of hematogenous spreading from primary cervical lesions.

Current assumptions are that actinomycosis can appear in both healthy individuals and immunocompromised hosts [3, 6]. Albeit it has been described in patients with HIV [8, 9], primary immunodeficiency [10], haematological malignancies [11], and individuals under anti-tumour necrosis factor (TNF) agents [7, 12], uncertainty remains about which host mechanisms are involved in controlling the infection and if the incidence of actinomycosis is increased in immunocompromised patients. In this case, we consider that the subacute onset and progression of the disease in weeks from a single lesion to multiple disseminated abscesses can possibly be related to the immunosuppression status of the patient, although there have been no reported cases of actinomycosis complicating treatment with azathioprine and mesalazine.

The gold standard for diagnosis of actinomycosis is isolation and culture from a usually sterile body site. The success of isolation in culture is reduced either by inhibition of growth of *Actinomyces* by copathogens like oral facultative pathogens (*Aggregatibacter actinomycetemcomitans, Propionibacterium propionicum, Streptococcus viridans* group), Gram-positive anaerobic cocci and Gram-negative anaerobic rods, short-term incubation period, or inadequate culture conditions. In a series of *Actinomyces* specimens, only 4.5% were isolated alone in culture, with *Actinomyces israelii* being the most frequently isolated species [4]. Coinfection by more than one *Actinomyces* is also documented, although with lower frequency [4]. In our case, *A. bovis* was isolated only after 14 days of incubation and confirmed by matrix-assisted laser desorption ionization time-of-flight (MALDI-TOF) mass spectrometry (MS). One possible explanation for its isolation alone is the previous antibiotic exposure, which could have limited the growth of some of the usual concomitant microorganisms. The fact that *A. bovis* was identified in only one of the multiple samples collected also suggests an effect of the previous antimicrobial courses, namely with tetracyclines (fluoroquinolones have poor or no activity over *Actinomyces* spp.) [3, 5]. The use of MALDI-TOF MS for pathogen identification supports the diagnosis, considering its high sensitivity [3]. Histological examination is not a reliable diagnosis method, considering that the pathognomonic sulfur granules are often difficult to identify or distinguish from other types of granules [4].

*Actinomyces bovis* is a rare isolate from the *Actinomyces* genus. There are a limited number of cases reported regarding this particular organism causing human infections, moreover in immunocompromised hosts (Table 1). Some authors consider that *A. bovis* is an exclusive animal pathogen and has been misidentified through history, corresponding in fact to the human species *A. israelii* [4]. Currently available diagnostic methods as MALDI-TOF MS have a significant sensitivity, being, therefore, more reliable than previous manual operant-dependent techniques.

**Table 1:** Published cases of human infection by *Actinomyces bovis*: review of the literature

Case [Reference]	Age/gender	Comorbidities	Immune condition	Diagnosis	Identification method	Treatment	Prognosis
1 [23]	24/M	None	Immunocompetent	Mitral valve endocarditis	Culture isolation	None (penicillin not available)	Death
2 [24]	41/M	None	Immunocompetent	Left chest wall mass and pericarditis	Culture isolation	Penicillin and abscess aspiration	Treated successfully
3 [25]	43/M	Hereditary haemorrhagic telangiectasia	Immunocompetent	Left temporal brain abscess	Culture isolation	Penicillin G + Sulphonamides and craniotomy with abscess excision	Treated successfully, mild personality change
4 [26]	43/F	Rheumatic heart disease	Immunocompetent	Mitral valve endocarditis	Culture isolation	Penicillin	Treated successfully
5 [27]	35/M	None	Immunocompetent	Left chest abscess and constrictive pericarditis	Culture isolation	Penicillin and pericardiectomy	Treated successfully
6 [10]	18/M	Common variable immunodeficiency	Immunodeficient	Cutaneous infection	Culture isolation	Penicillin	Treated successfully

*M* male, *F* female

*Actinomyces* are susceptible to several antibiotic agents in vitro [13]. Parenteral penicillin G is the first choice for treating actinomycosis; in adults, the usual dosage is 12–24 million units daily (18–24 million units daily for the thoracic form) for 2–6 weeks, followed (after clinical improvement) by oral penicillin V or amoxicillin for 6–12 months. These high dosages and prolonged duration of treatment are due to the characteristics of the infection, namely induration of infected tissue and lack of adequate blood supply in the surrounding area, with consequent poor antibiotic penetration into the tissue [3, 13]. Nevertheless, there is some evidence considering shorter antibiotic regimens with penicillins with beta-lactamase inhibitors with positive results [14]. Possible coinfection with Gram-positive and Gram-negative anaerobes, including *Enterobacteriaceae*, may require associations with metronidazole or aminoglycosides. Adjunctive interventional radiology procedures or surgical treatment may be considered, in order to reduce the inoculum (i.e. abscess drainage, bone curettage) or due to complications (i.e. necrotic tissue, fistulas, sinus tracts, or absence of clinical response to antimicrobial therapy) [3]. Although there are established international breakpoints for susceptibility testing for Gram-positive anaerobes [15] and evidence that susceptibility may change according to the identified species [13], it is not regularly performed. Susceptibility testing of *Actinomyces* spp. was not performed in this case, due to laboratory constraints and positive clinical response to treatment with benzylpenicillin.

There is no recommendation for the management of concomitant immunosuppressive treatment. Therefore, after discussion with the gastroenterology team, we started the antibiotic treatment without interrupting treatment with azathioprine and mesalazine. Azathioprine is a purine analogue that competitively inhibits the biosynthesis of purine nucleotides, widely used for the treatment of both Crohn's disease and ulcerative colitis.

It is almost entirely metabolised to 6-mercaptopurine (MP) and then to thioguanine nucleotides (TGNs), which are primarily responsible for both cytotoxic potential and the immunosuppressive effect of these agents. TGNs will have a cytotoxic effect on leucocytes by interfering with DNA synthesis [16, 17]. Moreover, T-cell apoptosis due to inhibition of the Ras-related C3 botulinum toxin substrate 1 (Rac1) is assumed [18]. There are some considerations regarding the involvement of genetic polymorphisms of thiopurine *S*-methyltransferase (TPMT), an enzyme involved in MP metabolism, and its reduced activity in mechanisms of augmented toxicity of azathioprine, namely myelosuppression and leukopenia [16]. Patients exposed to thiopurine therapy had increased risks of opportunistic infections compared with patients unexposed to thiopurines, although no actinomyces infection has been described in a case series [19].

Two cases of *Actinomyces* infection in patients receiving azathioprine are published in the literature, one in a patient treated with azathioprine and prednisolone for rheumatoid arthritis [20] and the second case in a renal transplant recipient, under cyclosporine A, azathioprine, and prednisolone triple therapy immunosuppression [21]. In both cases, the authors do not specify if the immunosuppressive agents were discontinued or substituted during antibiotic treatment. Considering the limited number of cases described, *Actinomyces* spp. infection occurring in patients receiving azathioprine appears to be an extremely uncommon event and, so far, there are no clinical reports of actinomycosis in patients receiving both azathioprine and 5-ASA for inflammatory bowel disease.

Mortality associated with actinomycosis ranges between 0 and 28%, with higher rates amongst CNS disease [5, 22]. The prognosis is favourable if the infection is early recognized and treated properly. Considering *Actinomyces* as a possible etiology may decrease costly unnecessary time-consuming investigations and redundant therapies. Careful follow-up is required, especially in immunocompromised patients, in order to promptly recognize and manage possible relapses.

Actinomycosis is nowadays an uncommon diagnosis, especially in developed countries. In clinical practice, it can be often a challenge, owing to the non-specific clinical and pathological features and its intrinsic difficulty in in vitro cultivating and intricate histological characteristics. However, considering the increasing number of immunocompromised patients worldwide and the various reports of infection by *Actinomyces* in this setting, actinomycosis should therefore be seen as a possible opportunistic infection in the context of immunosuppression. In addition, the identification of *A. bovis* as a pathogen in this case adds into consideration its role as a rare, but possible pathogen in humans.

## Data Availability

Not applicable.

## Abbreviations

5-ASA: 5-Aminosalicylic acid
CNS: Central nervous system
CT: Computed tomography
HIV: Human immunodeficiency virus
MALDI-TOF: Matrix-assisted laser desorption ionization time-of-flight
MP: Mercaptopurine
MS: Mass spectrometry
PCR: Polymerase chain reaction
PET: Positron emission tomography
TGN: Thioguanine nucleotide
TNF: Tumour necrosis factor
TPMT: Thiopurine S-methyltransferase
